# Older Adults Exhibit Greater Visual Cortex Inhibition and Reduced Visual Cortex Plasticity Compared to Younger Adults

**DOI:** 10.3389/fnins.2019.00607

**Published:** 2019-06-12

**Authors:** Dania Abuleil, Daphne L. McCulloch, Benjamin Thompson

**Affiliations:** School of Optometry and Vision Science, University of Waterloo, Waterloo, ON, Canada

**Keywords:** aging, binocular rivalry, visual evoked potential, cortical potentiation, visual cortex plasticity

## Abstract

Recent evidence indicates that inhibition within the visual cortex is greater in older than young adults. Increased inhibition has been associated with reduced visual cortex plasticity in animal models. We investigated whether age-related increases in human visual cortex inhibition occur in conjunction with reduced visual cortex plasticity. Visual cortex inhibition was measured psychophysically using binocular rivalry alternation rates (AR) for dichoptic gratings. Slower ARs are associated with a greater concentration of the inhibitory neurotransmitter GABA within the human visual cortex. Visual cortex plasticity was measured using an established paradigm for induction of long-term potentiation (LTP) -like increases in visually evoked potential (VEP) amplitude. Following rapid visual stimulation, greater increases in VEP amplitude indicate greater visual cortex plasticity. The study involved two groups; young (18–40 years, *n* = 29) and older adults (60–80 years, *n* = 18). VEPs were recorded for a 1 Hz onset/offset checkerboard stimulus before and after 9 Hz visual stimulation with the same stimulus. ARs were slower in older than young adults. In contrast to most previous studies, VEP amplitudes were significantly reduced following the rapid visual stimulation for young adults; older adult VEP amplitudes were unaffected. Our AR results replicate previous observations of increased visual cortex inhibition in the older adults. Rapid visual stimulation significantly altered VEP amplitude in young adults, albeit in the opposite direction than predicted. VEP amplitudes did not change in older adults suggesting an association between increased inhibition and reduced plasticity within the human visual cortex.

## Introduction

Visual cortex plasticity is high during an early “critical period” of development and then gradually declines as the brain matures. Within animal models, the closure of the critical period has been associated, in part, with an increase in the concentration of gamma aminobutyric acid (GABA) (Fagiolini and Hensch, 2000; Fagiolini et al., 2004; Sale et al., 2010), an inhibitory neurotransmitter. In addition, critical periods for visual cortex characteristics such as ocular dominance can be reopened in mature, post-critical period animals through pharmacological, or environmental manipulations that reduce GABA concentration (Sale et al., 2007; Vetencourt et al., 2008; Harauzov et al., 2010). Therefore, the current evidence from animal models strongly suggests that GABA concentration plays a key role in modulating visual cortex plasticity.

Less is known about the time course of visual cortex plasticity in humans; however, there is evidence that plasticity is high in early childhood and declines with increasing age (Sale et al., 2010; Siu et al., 2017) as is the case in animal models. For example, the response to patching therapy decreases with age in children with amblyopia (Holmes, 2011). Amblyopia is a neurodevelopmental disorder of the visual cortex and recovery of vision in an amblyopic eye requires substantial visual cortex plasticity. In addition, as predicted by animal models, GABA may play a role in regulating human cortical plasticity. Magnetic resonance spectroscopy (MRS) studies have shown that the neuromodulation technique anodal transcranial direct current stimulation (a-tDCS) reduces GABA concentration in the adult motor cortex and that larger GABA reductions are associated with increased motor learning (Stagg et al., 2011; Patel et al., 2017). A-tDCS can also temporarily improve vision in adults with amblyopia, perhaps by modulating GABA concentration (Spiegel et al., 2013a, 2013b; Ding et al., 2016; Castaño-Castaño et al., 2017).

Human cortical GABA levels continue to change in adulthood, and these changes may influence visual cortex plasticity. A number of MRS studies have reported a general decrease in GABA concentration within the frontal and parietal regions of the human brain from age 20 to 76 years (Gao et al., 2013; Porges et al., 2017) that may be related to age-related cognitive decline (Hua et al., 2008; Porges et al., 2017). However, these studies did not measure visual cortex GABA concentrations. In contrast, a recent MRS study specifically targeting the primary visual cortex found significantly higher GABA levels in older (63–78 years old) relative to younger (20–34 years old) adults (Pitchaimuthu et al., 2017). This result is consistent with gene expression studies in primates (Liao et al., 2016), humans (Pinto et al., 2010), and adult primate V1 neurophysiological recordings (Wang et al., 2018), all suggesting an increase in GABA-mediated visual cortex inhibition in older relative to younger adults.

A number of psychophysical observations also provide evidence for increased GABA mediated inhibition in older adults (Karas and McKendrick, 2009, 2011, 2012; Nguyen and McKendrick, 2016; Kondo and Kochiyama, 2017; Wang et al., 2018). For example, binocular rivalry alternation rates (AR) are slower in older than in younger adults (Jalavisto, 1964; Ukai et al., 2003; Kondo and Kochiyama, 2017; Pitchaimuthu et al., 2017). Binocular rivalry occurs when dissimilar images are shown to each eye. This causes perceptual alternation between each eye’s image. Several independent MRS studies have demonstrated that binocular rivalry AR are negatively associated with visual cortex GABA concentration (van Loon et al., 2013; Fesi and Mendola, 2015; Pitchaimuthu et al., 2017). Therefore, slowed AR in older adults indicate an increase in GABA mediated inhibition with age.

The aim of this study was to investigate whether age-related changes in adult human visual cortex GABA concentration influences visual cortex plasticity. Visual cortex inhibition was measured using binocular rivalry AR (van Loon et al., 2013; Fesi and Mendola, 2015; Kondo and Kochiyama, 2017; Pitchaimuthu et al., 2017; Keute et al., 2019). Plasticity was measured indirectly using an established technique for inducing long term potentiation (LTP) -like plasticity within the human visual cortex (Teyler et al., 2005; Normann et al., 2007; Ross et al., 2008; de Gobbi Porto et al., 2015; Klöppel et al., 2015; Spriggs et al., 2017). The technique involves the measurement of visually-evoked potentials (VEPs) directly before and after a period of rapid visual stimulation (tetanization). VEP amplitudes are increased by visual tetanization and the magnitude of the increase provides an index of visual cortex plasticity (see Sanders et al., 2018 for a comprehensive review). Our hypothesis was that older adults would exhibit greater visual cortex inhibition (slower binocular rivalry AR) and reduced visual cortex plasticity (reduced VEP amplitude modulation in response to visual tetanization). This pattern of results would be consistent with an association between an age-related increase in visual cortex GABA concentration and reduced visual cortex plasticity.

## Materials and Methods

### Participants

Thirty young adults (mean age 26 years, range 20–39, and 19 female) and 20 older adults (mean age 70, range 61–80, and 16 female) with normal or corrected-to-normal vision, no eye disease, and good health participated in the study. Stringent exclusion criteria for older adults were applied. These included any diagnosed or self-reported vision disorder, Parkinson’s disease, Huntington’s disease, and multiple sclerosis, as well as learning disabilities. Participants taking psychoactive medications were excluded. All participants were informed of the nature of the study before participation and provided written informed consent. The project was approved by the University of Waterloo Research Ethics Committee.

### Binocular Rivalry Measurements

Orthogonally oriented (45° and 135°) sinusoidal gratings [0.5 cycles per degree (cpd), subtending 6.1 degrees of visual angle] were presented dichoptically via red/green anaglyphic glasses on a 24″ Asus^®^ 3D monitor. The luminance and contrast of the gratings were matched using a Chroma Meter CS-100^®^ spectrophotometer. Stimuli were viewed from 57 cm using a chin rest. Participants reported red grating, green grating or piecemeal percepts using a computer keyboard. Alternations were defined as any change in percept (e.g., left eye to right eye, right eye to piecemeal etc).

Six 60-s trials were presented with 1-min breaks between each trial. Red/green glasses were worn with the red lens over the right eye for the first 3 trials and reversed for the last 3 trials.

### Electrophysiological Measurements

A clinical-grade EEG system (Espion E2^®^ electrophysiological testing system version 5.2, Diagnosis, United States) was used to measure VEPs. Three active channels – Oz, PO7, and PO8 referenced to Fz (based on the 10–20 system) – were recorded. These positions are where previous LTP-like effects have been found. A ground electrode was placed on the right ear. Individual recordings with artifacts such as blinks or other high voltage events (±30 μV) were rejected from the average using real-time artifact rejection software (Espion E2). Waveforms were sampled at a frequency of 1000 Hz with a band pass filter of 0.3–100 Hz.

**FIGURE 1:**
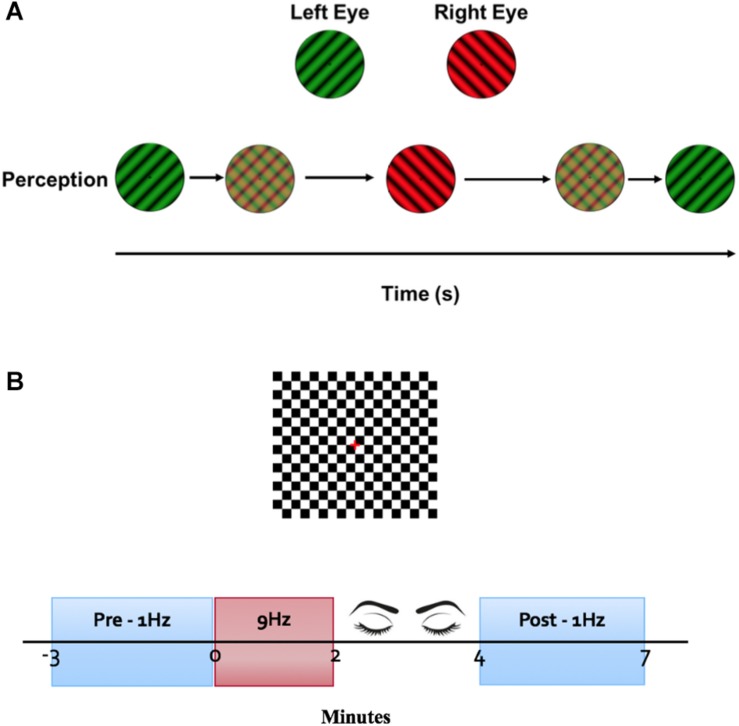
Experimental protocol. **(A)** Binocular rivalry stimulus. A luminance-matched dichoptic grating was presented on a black background. Participants wearing red/green glasses used a keyboard to report perception of stimulus. **(B)** VEP stimulus. Onset and offset of a black and white checkerboard was presented on a luminance-matched gray background. VEPs were averaged for 3 min at a stimulus frequency of 1 Hz as pre and post measurements. Immediately following the baseline VEP measurement, a tetanization block of stimuli presented at 9 Hz was presented for 2 min. Participants closed their eyes for 2 min before the post tetinization VEPs were recorded.

The LTP-like induction protocol described by Teyler et al. (2005) was adapted and applied to the clinical system. A checkerboard stimulus (0.3 degree check width), with a square field of 7.7° was presented on a 60 Hz CRT screen at 1.5 m. The checkerboard was presented with a 400 ms onset and 600 ms offset at a frequency of 1 Hz for 3 min for both pre and post tetanization. Immediately following the pre measure, the same checkerboard was presented on/off (on for 30 ms, off for 80 ms) at a frequency of 9 Hz for 2 min. Following tetanization, participants closed their eyes for 2 min. The post measure was then recorded (Figure 1).

**FIGURE 2:**
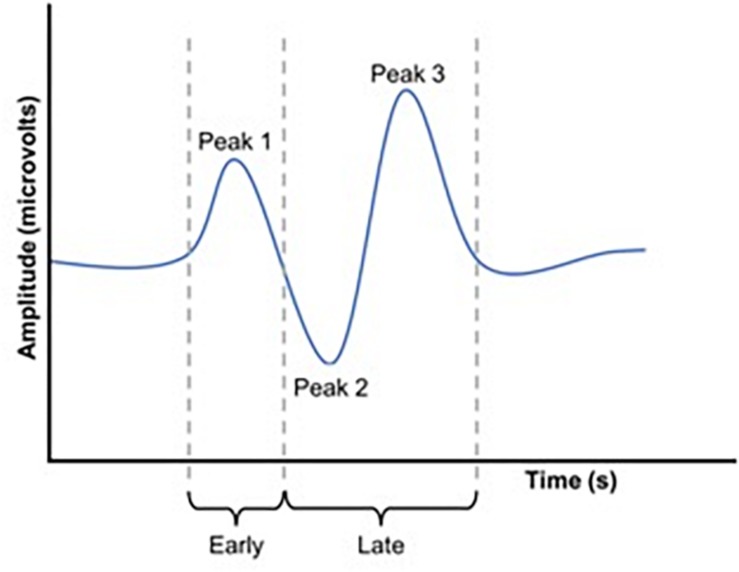
Schematic representation of a VEP waveform. RMS values were calculated for both an early and a late window. These windows were set to capture P1 (early window) and P2 and P3 (late window).

**FIGURE 3:**
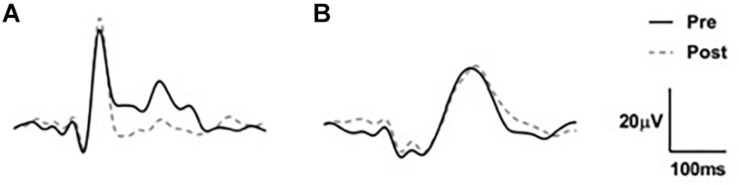
Two examples of VEP waveforms **(A)** n older adult and **(B)** young adult averaged across channels demonstrating the individual variation between participant VEPs.

**FIGURE 4:**
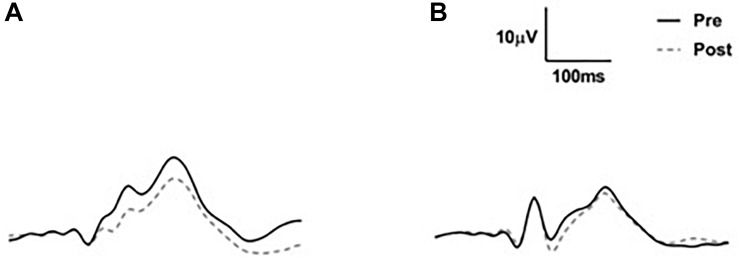
Group average VEP waveforms in microvolts. **(A)** Average VEP waveforms across all three electrodes for the young adult group. **(B)** Average VEP waveforms across all three electrodes for the older adult group. Note that there was considerable between-subject variability (not shown).

Waveforms were exported from the VEP system as text files, low pass filtered at 30 Hz using EEGlab in Matlab (2016a) and analyzed manually (Figure 2). Because we used a central-field on-off checkerboard stimulus, the initial polarity of the resulting waveform varied across participants due to individual differences in cortical anatomy (Halliday and Michael, 1970; Michael and Halliday, 1971; Jeffreys and Axford, 1972; Emmerson-Hanover et al., 1994; Figure 3). Root-mean-square (RMS) contrasts were used for analysis because they are insensitive to waveform polarity. To perform the RMS analysis, we first identified three peaks within each pre-visual tetanization waveform. The first prominent deflection from baseline, whether positive or negative was labeled peak 1. Peak 2 and peak 3 were labeled as the following deflections, respectively. The average latency of each peak was calculated and a window for calculating RMS contrast was centered around each peak. We opted for the convention of using windows based on average latencies because inter-individual differences in waveform precluded any other approach to the analysis. The maximum possible window size without overlap was chosen symmetrically around each peak (peak 1, 64–102 ms; peak 2, 103–138 ms; and peak 3: 139–172 ms). The window around peak 1 was labeled the early period, while the peak 2 and peak 3 windows were combined and labeled the late period. The two latter peaks were combined because they overlapped in approximately 30% of the younger participants. Combining the peaks enabled a fair comparison between age groups. Measurements were taken for each channel (electrode position) individually. RMS contrast measurements were averaged across channels for each individual participant (Figure 4).

VEP data for one young adult were not obtained due to technical difficulties. VEP waveforms for two older adult participants had low signal-to-noise and were also excluded from analysis. Therefore, 29 young and 18 older adult datasets were included in the final VEP analyses.

### Statistical Analysis

For the binocular rivalry alternation rate measurement, an independent *t*-test was used to assess differences between groups.

To test for group differences in LTP-like changes in VEP RMS contrast, a mixed ANOVA with factors of Group (young vs. older), and Time (pre vs. post tetanization) was conducted on the VEP RMS values. RMS values were averaged across electrodes. ANOVA analyses were conducted for the early and late periods separately because previous studies have reported VEP amplitude potentiation that is highly specific to particular timepoints within the waveform (Teyler et al., 2005; Normann et al., 2007). *Post hoc* testing of significant interactions was conducted using *t*-tests.

A linear regression analysis with a dependant variable of AR and dependant variable of Change in VEP RMS Contrast (post tetanization minus pre tetanization) was conducted for each age group separately to test for any within group associations between AR and LTP-like changes in VEP RMS contrast.

## Results

### Group Differences in Alternation Rates

**FIGURE 5:**
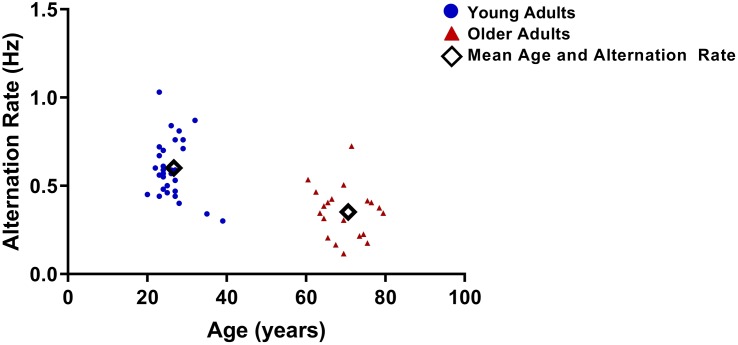
Alternation rates (AR) for each participant vs. age. AR were significantly lower for older adults.

As reported in a previous study (Ukai et al., 2003), older-adults experienced significantly slower AR (mean = 0.33 ± 0.14 alternations/second) than did young adults (mean = 0.59 ± 0.15 alternation/second; *t*_48_ = 5.5, *p* < 0.001; Figure 5). There was no significant difference between age groups for time spent in piecemeal: young mean = 10.84 (*SD* = 7.85); older mean = 9.26 (*D* = 7.81); *t*_48_ = 0.699, *p* = 0.488.

### Group Differences in Visual Cortex LTP-Like Plasticity

For the early period, there was no significant difference between age groups (*F*_1,45_ = 0.2, *p* = 0.7) or two-way interaction between time and group (*F*_1,45_ = 2.1, *p* = 0.2). The main effect of time was also not significant (*F*_1,45_ = 0.5, *p* = 0.5).

**FIGURE 6:**
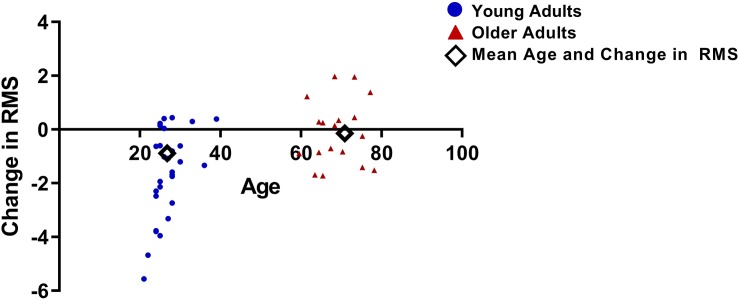
Difference in RMS values between pre and post tetanization for young and older adults, calculated as the change in pre to post RMS for the late period.

For the late period, a significant two-way interaction was observed between time and group (*F*_1,45_ = 10.8, *p* = 0.002; Figure 6). A significant main effect of time was also observed (*F*_1,45_ = 15.6, *p* < 0.001) and there was no significant main effect of group (*F*_1,45_ = 1.7, *p* = 0.2). *Post hoc t*-tests conducted on the late period data for each group separately indicated a significant reduction in VEP amplitude from pre to post tetanization for the young adult group (*t*_28_ = 5.3, *p* < 0.001) but not for the older adult group (*t*_17_ = 0.5, *p* = 0.6).

A regression analysis with AR as the dependent variable and change in late period RMS (post RMS – pre RMS) as the independent variable revealed no association between change in late period RMS and AR for either the young (*B* = -0.01, *SE* = 0.02, *p* = 0.6) or the older adult groups (*B* = -0.01, *SE* = 0.03, *p* = 0.7).

## Discussion

The aim of this study was to investigate whether the increase in visual cortex inhibition that occurs from young to older adulthood is associated with a reduction in visual cortex plasticity. The slower AR that we observed in our older adult group supports previous work (Ukai et al., 2003; Norman et al., 2007; Kondo and Kochiyama, 2017; Arani et al., 2018) and is consistent with increased GABA-mediated visual cortex inhibition (van Loon et al., 2013; Pitchaimuthu et al., 2017; Kondo et al., 2018). In agreement with our hypothesis, the young adult group exhibited greater changes in VEP amplitude in response to rapid visual stimulation than the older adult group, indicative of greater visual cortex plasticity. However, the change in VEP amplitude was opposite to that reported in most previous literature – a depotentiation rather than a potentiation.

Previous animal studies have suggested that GABA-mediated visual cortex inhibition is reduced in older adulthood (Leventhal et al., 2003; Hua et al., 2008). However, recent MRS measurements in humans have demonstrated a clear reduction in visual cortex GABA concentration in older compared to younger adults; this is associated with lower binocular rivalry AR in the older group. Our results support this previous work and extend the results to demonstrate that visual cortex plasticity is also reduced in the older adult group. This finding is in agreement with animal neurophysiology indicating that increased GABA-mediated inhibition reduces primary visual cortex plasticity (Sale et al., 2007; Vetencourt et al., 2008; Harauzov et al., 2010).

Surprisingly, young adults showed significant VEP depotentiation rather than potentiation following visual tetanization. This contrasts with most prior studies (Teyler et al., 2005; Normann et al., 2007; Ross et al., 2008; de Gobbi Porto et al., 2015; Klöppel et al., 2015), although depotentiation of the BOLD response following visual tetanization has been reported in young adults (Lahr et al., 2014). Additionally, some studies observed considerable variability across participants in the response to visual tetinization for both young and older adults (de Gobbi Porto et al., 2015; Klöppel et al., 2015). Our results suggest that the effect of rapid visual stimulation on VEP amplitude may not generalize across individuals or specific stimulus parameters. The protocols of Teyler and Normann’s groups, which reported potentiation in young adults, differed in their stimulus protocols from our current study. The former used hemifield onset:offset checkerboard stimuli; the later used a full-field checkerboard reversal. Although we used similar check stimuli, we employed central field stimulation with onset:offset presentations of pre, post, and tetanization stimuli. Our observation of reduced VEP amplitudes following rapid visual stimulation may be indicative of long-term depression or adaptation within the visual cortex. Plasticity within the visual cortex may include both potentiation and depotentiation effects. Thus, either an increase or decrease in VEP amplitude following tetanization reflects plasticity toward greater potentiation or depotentiation, respectively. Although stable VEP amplitudes following tetanization do not rule out equal potentiation and adaptation effects, we hypothesize that stable amplitudes more likely reflect lower overall levels of plasticity.

Older adults showed no significant change in VEP amplitude following rapid visual stimulation. This is consistent with an increased level of visual cortex inhibition associated with reduced plasticity. One previous study has reported successful LTP-like induction in older adults using visual tetanization (de Gobbi Porto et al., 2015). However, another study has reported reduced LTP-like induction in older adults compared to young adults (Spriggs et al., 2017), in general agreement with our results.

Our regression analysis revealed no significant association between AR and LTP-like changes within each age group. One explanation for this result is that our indirect measures of GABA concentration and visual cortex plasticity do not have strong enough correlations with the actual state of the visual cortex to reveal subtle within-age-group associations (Chan et al., 2018; Keute et al., 2019). It is also possible that other neural mechanisms aside from visual cortex GABA concentration may be involved. For example, a recent study suggests that neural noise may play a stronger role in the association between slower AR and age than an increase in neural inhibition (Arani et al., 2018).

Our study has a number of limitations. These include participants in each group not being matched with respect to education levels, IQ or gender. In addition, our sample size may have prevented us from detecting subtle associations between variables within our regression analysis (Pitchaimuthu, 2017).

In conclusion, age-group differences in AR and the change in VEP amplitude in response to rapid visual stimulation indicate increased inhibition and reduced plasticity within the visual cortex of older adults. The findings have implications for the treatment of brain-based visual disorders such as amblyopia in adulthood.

## Ethics Statement

This study was carried out in accordance with the recommendations of the Office of Research Ethics at the University of Waterloo with written informed consent from all subjects in accordance with the Declaration of Helsinki.

## Author Contributions

All authors were involved in study planning and design, data analysis, and editing of the manuscript.

## Conflict of Interest Statement

The authors declare that the research was conducted in the absence of any commercial or financial relationships that could be construed as a potential conflict of interest.
